# Microorganisms in *Ixodes ricinus* ticks in southern and southwestern Norway

**DOI:** 10.1016/j.ijppaw.2026.101252

**Published:** 2026-06-12

**Authors:** Arnulf Soleng, Deepa Gurung, Vivian Kjelland, Katrine M. Paulsen, Benedikte N. Pedersen, Erik G. Granquist, John H.-O. Pettersson, Rose Vikse, Snorre Stuen, Åshild K. Andreassen

**Affiliations:** a Department of Virology, Division for Infection Control and Environmental Health, Norwegian Institute of Public Health, Oslo, 0456, Norway; b Department of Natural Sciences, Faculty of Engineering and Science, University of Agder, Kristiansand, 4630, Norway; c Department of Production Animal Clinical Sciences, Faculty of Veterinary Medicine, Norwegian University of Life Sciences, 1433 Ås, Sandnes, 4325, Norway; d Department of Microbiology, Swedish Veterinary Agency, Uppsala, 751 89, Sweden; e Zooonosis Science Center, Department of Medical Sciences, Uppsala University, Uppsala, 752 37, Sweden

**Keywords:** *Anaplasma*, *Borrelia*, Louping-ill virus, Tick-borne encephalitis virus, *Wolbachia*, *Midichloria*

## Abstract

Tick-borne diseases constitute a major group of emerging vector-borne infections and represent a growing public health concern in many European countries. In Europe, the most clinically significant agents transmitted by *Ixodes ricinus* are tick-borne encephalitis virus (TBEV) and *Borrelia burgdorferi* sensu lato, the causative agents of tick-borne encephalitis and Lyme borreliosis, respectively. *Ixodes ricinus* also serves as a vector for other pathogenic microorganisms, including louping-ill virus (LIV) and *Anaplasma phagocytophilum.* Various endosymbionts, such as *Wolbachia pipientis* and *Midichloria mitochondrii*, are also found in ticks. In this study, we assessed the prevalence of TBEV, LIV, *B. burgdorferi* s. l., *A. phagocytophilum*, *W. pipientis*, and *M. mitochondrii* in 3437 *I. ricinus* ticks collected from five locations in Norway. The estimated pool prevalence of TBEV ranged from 0 to 0.4% in nymphs, while the prevalence in adults ranged from 0 to 7.1%. In contrast, no LIV was detected. *Borrelia burgdorferi* s. l. was detected at all sites, with prevalences ranging from 3.4% to 14.3% (overall prevalence of 5.7%), representing four genotypes (*B. afzelii*, *B. garinii*, *B. valaisiana* and *B. burgdorferi* sensu stricto). Estimated pooled prevalence of *A. phagocytophilum* was 5.1% in nymphs, while the overall prevalence in adults was 19.8%. In adult ticks, *W. pipientis* and *M. mitochondrii* occurred at overall prevalences of 11.0% and 84.1%, respectively. In only 12.3% of ticks, none of the agents investigated were detected. However, 32.6% of the ticks showed co-occurrence of infectious agents and/or the endosymbionts. Thus, this underscores the need for improved understanding of microbial interactions influencing tick-borne disease outcomes.

## Highlights

• Norwegian *Ixodes ricinus* ticks carried diverse pathogens and endosymbionts
• TBEV was rare, and louping-ill virus was not detected
• *Borrelia*, *Anaplasma*, *Midichloria*, and *Wolbachia* were frequently detected
• Co-occurrences were common, indicating complex microbial interactions

## Graphical abstract

**Figure:**
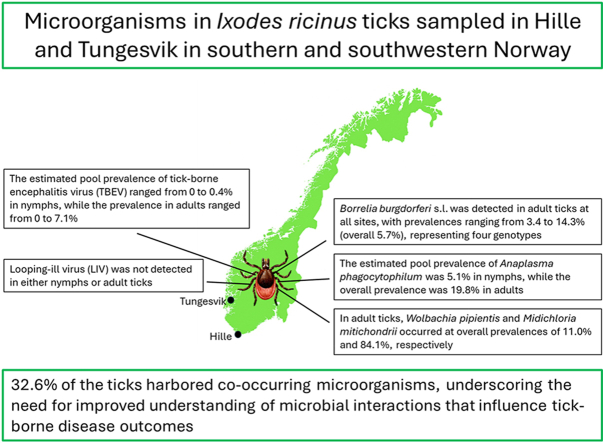

## 1 Introduction

Ticks are significant vectors of pathogens that impact human and animal health globally. The list of known or potential pathogens is constantly evolving, and includes a variety of viruses, bacteria, and parasites afflicting humans and animals worldwide (de la Fuente et al., 2008; Heyman et al., 2010; Dantas-Torres et al., 2012; Rizzoli et al., 2014). Ticks may also host a diverse range of commensal and symbiotic microorganisms (Bonnet et al., 2017). Such microorganisms might be important to tick ecology, pathogen transmission and/or establishment of infections. However, the biology of these microorganisms and their effects remain understudied and overlooked (Bonnet et al., 2017). Nevertheless, it is suggested that endosymbiotic bacteria like *Wolbachia pipientis* might function as a biological control agent of vectors and their diseases (LePage and Bordenstein, 2013; Skinner et al., 2022). Endosymbionts, especially the rickettsia species, are passed vertically by transovarial transmission from female to eggs and modify host biology in numerous ways, including inducing reproductive manipulations such as feminization, parthenogenesis, male killing, and sperm–egg incompatibility (Rymaszewska, 2007; Parola et al., 2013; Ravindran et al., 2023)**.** They can also spread horizontally across species, contributing to their widespread, global distribution among diverse invertebrate hosts (Werren et al., 2008).

In Norway the multi-competent tick vector *Ixodes ricinus* is the dominant tick species affecting human and animal health. Over the last few decades research in Norway has acquired new knowledge on the distribution and impact of *I*. *ricinus* ticks and their associated pathogens (Kjelland et al., 2010, 2018; Andreassen et al., 2012; Soleng and Kjelland, 2013; Hvidsten et al., 2014, 2020; Larsen et al., 2014; Soleng et al., 2018; Jenkins et al., 2019; Paulsen et al., 2015, Paulsen et al., 2018a, Paulsen et al., 2018b; 2019; Kjær et al., 2019a, b, 2020; Pedersen et al., 2019a, b; Vikse et al., 2020; Lamsal et al., 2023, b; Lamsal et al., 2025; Maharjan et al., 2025). However, the knowledge about commensal and symbiotic microorganisms in ticks in Norway is limited. In general, the origin of *Wolbachia* in ticks, and the effects on tick biology, is unknown. *Wolbachia* is known to be genetically diverse in invertebrates (Werren et al., 2008). To date, no studies have detected this bacterium in ticks in Norway (Granquist et al., 2014)*. Midichloria mitochondrii* is known to be the dominant bacterium in the microbial community of *I. ricinus* (Sassera et al., 2006; Epis et al., 2013). For example, Granquist et al. (2014) found a prevalence of 35.1% among adult ticks collected on the Norwegian west coast as well as a significant geographical variation in prevalence among tick nymphs.

The aim of the present study was to examine nymphal and adult *I. ricinus* ticks for the presence or absence of tick-borne encephalitis virus (TBEV), louping-ill virus (LIV) and selected bacterial pathogens and commensal/symbiotic microorganisms. Furthermore, the study aimed to elucidate if co-infections of these pathogens and co-occurrence of other microorganisms are common in these ticks. The investigation was conducted at five locations where ticks are known to be abundant: three locations in the southwestern part of Norway, and two locations on an island in southern Norway.

## 2 Materials and methods

### 2.1 Collection of ticks

Adult and nymphal *I. ricinus* ticks were collected from five locations in Norway (Table 1). Location 1, 2 and 3 in Tungesvik is located on the southwestern coast of Norway. The area is a farmland with grazing domestic cattle (*Bos taurus*) and sheep (*Ovis aries*). The vegetation is dominated by deciduous trees, bushes and grass. Location 4 and 5 are located on the Island Hille on the southern coast. This island has a high density of roe deer (*Capreolus capreolus*) and some grazing cattle with varying degree of deciduous trees, bilberry, shrubs and ferns.

**Table 1:** Tick sampling locations and year of sampling. All samplings took place in June. Location number 1, 2 and 3 are situated on the southwestern coast of the Norwegian mainland. Location number 4 and 5 are situated on the island Hille on the southernmost part of Norway.

Location (year)	County	Coordinates (Lat/Lon^a^)	Elevation (masl^b^)	Distance from the sea (meters)	Nymphs (n)	Adults (n) [male/female]
Tungesvik 1 (2014)	Vestland	59.7338272/5.9930412	10–50	10–200	310	21 [10/11]
Tungesvik 2 (2014)	Vestland	59.7349399/5.9778644	20–50	15–150	540	29 [17/12]
Tungesvik 3 (2014)	Vestland	59.7345883/5.9774591	60–100	160–250	780	28 [14/14]
Hille 4 (2011)	Agder	58.0067584/7.3615383	25–60	5–150	460	52 [24/28]
Hille 4 (2013)	Agder	58.0067584/7.3615383	25–60	5–150	120	0
Hille 4 (2014)	Agder	58.0067584/7.3615383	25–60	5–150	740	42 [19/23]
Hille 5 (2011)	Agder	58.0113052/7.3532963	8–10	200–300	260	55 [29/26]

a Lat/Lon: Latitude/Longitude.

b masl: meters above sea level.

Questing ticks were collected in June in 2011, 2013 and 2014 by flagging the ground vegetation with a white terry cloth (1.0m × 0.7m) attached to a stick as described by Soleng and Kjelland (2013). Sampling was conducted on days when vegetation was dry. Adult ticks were individually isolated, while nymphs were pooled in groups of 10 in each tube. Larvae were not included in the study. The tubes with the ticks were stored on crushed ice in a cooling box during transportation to the laboratory, then stored at −80°C until further analysis.

### 2.2 Nucleic acid extraction

Pooled nymphs (10 nymphs per pool) and individual adult ticks were homogenized in FastPrep®-24 5G homogenizer (MP Biomedical Life Science, CA, USA). The FastPrep® tubes was added 350 μl and 400 μl of RLT lysis buffer (QIAGEN GmbH, Hilden, Germany) for nymphs and adults, respectively.

Total RNA was extracted from nymphs by the RNeasy mini kit in an automated QIAcube (both: QIAGEN Inc., Valencia, CA, USA), according to the manufacturers’ instructions. RNA was eluted by adding 60 μl 1 mM Tris-HCl buffer (pH 8.0).

Total nucleic acid (TNA) was extracted from adult ticks with MagNa Pure LC Total Nucleic Acid High Performance Isolation kit with a MagNa Pure LC 2.0 instrument (both: Roche Diagnostics GmbH, 82372 Penzberg, Germany), according to the manufacturers’ instructions. TNA was eluted by adding 60 μl elution buffer. The extracted RNA and TNA were stored at −80°C until further analysis.

### 2.3 Detection of microorganisms

For the detection of microorganisms, cDNA was used as template. The extracted RNA and TNA were reversed transcribed to cDNA by the High-Capacity cDNA Reverse Transcription Kit (Applied Biosystem, Foster city, CA, USA) with random primers according to the manufacturer's instructions, as previously described (Andreassen et al., 2012). Undiluted cDNA was used as template for the detection of TBEV, LIV, *W*. *pipientis* and *M*. *mitochondrii* by real-time PCR. For the detection of *A*. *phagocytophilum* and *B. burgdorferi* s. l. by real-time PCR, diluted (1:10) cDNA was used as template to avoid inhibition. This may lead to underreporting of infection prevalence. For the genotyping of *B. burgdorferi* s. l. undiluted cDNA was used as template.

Tick-borne encephalitis virus was detected by an in-house RT-real-time PCR assay, and positive samples were verified by pyrosequencing, as previously described by Andreassen et al. (2012) (Table 2.). Ticks collected at location 1, 2, 3 and 4 were analysed for TBEV – omitting location 5. Furthermore, at location 4 only the samples from June 2014 were analysed for TBEV. Thus, a total of 237 nymphs pools and 152 adult ticks were analysed.

**Table 2:** Primer and probe sequences used for detection of pathogens.

Primer name	Sequence(5’→3′)	Genome position	GenBank accession No.
TBE 320 Forward^1^	GGGAGCGCAAAACTG GAA	1662-1680	U27495.1
TBE 373 Rerverse^1^	TGAGGAGCCCCAAATTC AAC-biotin	1696–1715	U27495.1
TBE 339 probe^1^	(FAM)-AACGCAGAAAGAC-(MGB)	1681–1693	U27495.1
LIV 410 Forward^2^	CCAACAAAATAGTGTACACAGTGAAGGTTG	401–430	D12936.1
LIV 1000 Reverse^2^	CGGGTCAAAGCCATGCAGG	999–1017	D12936.1
LIV 680 probe^2^	(FAM)-AGCATGATGGAAACCCAC-(MGB)	683–702	D12936.1
RF forward primer^3^	GCTGTAAACGATGCACACTTGGT		
RF reverse primer^3^	GGCGGCACACTTAACACGTTAG		
LB probe^3^	6FAM-TTCGGTACTAACTTTTAGTTAA-MGBNFQ		
IGS 1 forward primer^4^	GTATGTTTAGTGAGGGGGGTG	2306–2326	U03396
IGS 1 reverse primer^4^	GGATCATAGCTCAGGTGGTTAG	3334–3313	
IGS 2 forward primer^4^	AGGGGGGTGAACTCGTAACAAG	2318–2339	
IGS 2 reverse primer^4^	GTCTGATAAACCTGAGGTCGGA	3305–3284	
ApMSP2 252F^5^	ACAGTCCAGCGTTTAGCA AGA		
ApMSP2 459R^5^	GCACCACCAATACCATAA CCA		
gyrB-MidiF^6^	CTTGAGAGCAGAACCAC CTA		
gyr-MidiR^6^	CAAGCTCTGCCGCCGAAA TATCTT		
Wolwsp81F^7^	TGGTCCAATAAGTGAAGA AAC		
Wolwsp691R^7^	AAAAATTAAACGCTACTC CA		

1-Andreassen et al. (2012), 2- Katrine M Paulsen et al. (2018a), 3-(Kjelland et al. (2010), 4-Bunikis et al. (2004), 5-Granquist et al. (2010), 6-Sassera et al. (2008), 7-Braig et al. (1998).

Louping-ill virus was analysed by an in-house RT-real-time PCR assay amplifying a 617 bp (nt 401-1017) fragment of the E-gene of LIV (Gene bank accession number D12936.1). The RT-real-time PCR conditions were as follows: 1 mM Qiagen One step-RT-PCR buffer (5x), 0.3 mM MgCl_2_, 0.4 mM dNTP, 0.4 μM forward primer (LIV 410F), 0.4 μM reverse primer (LIV 1000R), 0.3 μM probe (LIV 680 probe) (Table 2), 0.038 Units Pt-Taq (Invitrogen Life Technology, Inc., Carlsbad, CA, USA), RNase free Water (Sigma) was added up to 22 μl.

The PCR reaction was run in a total volume of 25 μl including 3 μl cDNA on a Rotor-Gene 6000 (Qiagen, Hilden, Germany) with the following cycling conditions: 1 cycle 95°C 2 min, 45 cycles 95°C 15 s, 54°C 30 s, 72°C 30 s. Each PCR run included the Norwegian louping-ill virus strain LI/NOR as positive control to determine the sensitivity of the method, and water was used as negative control (data not shown). All nymph pools (n = 321) and adult ticks (n = 227) were analysed for LIV.

*Borrelia burgdorferi* s. l. 16S RNA fraction was detected by StepOnePlus™ Real-Time PCR Systems (Applied Biosystems Inc., New Jersy, USA) and genotyped by Sanger sequencing on a 3130 Genetic Analyzer (Applied Biosystems Inc.) as previously described by Kjelland et al. (2010) (Table 2), with the following modification: cDNA was used as template in all reactions (1:10 diluted for the detection, and undiluted for the genotyping). Adult tick samples (n = 227) from all locations were analysed for the presence of *B*. *burgdorferi* s. l.

*Anaplasma phagocytophilum* was detected by SYBR Green real-time PCR as described by Granquist et al. (2010) (Table 2), with the following modification: 1:10 diluted cDNA was used as template. All samples were further analysed by melting-point analysis to confirm that alle were similar to the positive control. All nymph pools (n = 321) and adult ticks (n = 227) were analysed for *A. phagocytophilum*.

*Wolbachia pipientis* was detected by SYBR Green real time PCR with *wsp* primers described by Braig et al. (1998), in a Rotor-Gene 6000 thermal cycler (QIAGEN Inc.,Valencia, CA, USA) (Table 2). The PCR mixture consisted of Perfecta® SYBR® Green FastMix® (Quanta Biosciences Inc., Gaithersburg, USA), primers (0.4 μM) and the template cDNA. All adult ticks (n = 227) were analysed for *W*. *pipientis*.

*Midichloria mitochondrii* was detected by SYBR Green Real-Time PCR with *gyrB* primers described by Sassera et al. (2008) (Table 2), in a Rotor-Gene 6000 thermal cycler (QIAGEN Inc., Valencia, CA, USA). The PCR mixture consisted of SYBR® Green Master Mix (Thermo Fisher Scientific Inc., USA), primers (0.4 μM) and the template cDNA. All adult ticks (n = 227) were analysed for *M*. *mitichondrii.*

### 2.4 Statistical analysis

Prevalence of individually tested adult ticks was calculated as the proportion of positive specimens. The calculation of TBEV prevalence was only based on pyrosequencing confirmed positives. Exact 95% confidence intervals were calculated by the Clopper–Pearson method assuming a binomial distribution. Overall prevalence and corresponding 95% confidence intervals were calculated by aggregating all specimens and applying the same binomial approach.

For pooled nymph samples, prevalence was calculated as the estimated pool prevalence (EPP) with the Epitools epidemiological calculator (Sergeant, 2025). The minimum infection rate (MIR), defined as the number of positive pools divided by the total number of tested individuals (Andreassen et al., 2012). Exact 95% confidence intervals for EPP were calculated by deriving exact (Clopper–Pearson) confidence intervals for the proportion of positive pools and transforming these to individual-level prevalence. Overall prevalence estimates and corresponding confidence intervals for pooled nymphs were calculated by aggregating all samples and applying the same methods.

## 3 Results and discussion

The results are subject to several limitations, including limited sample sizes and restricted geographical coverage, comprising only two main areas with three and two sampling locations, respectively. Another limitation of this study is that sampling was conducted only once per year. As a result, the estimates may not capture seasonal variation in prevalence which is known to occur for several pathogens in ticks in southern Norway (Cotes-Perdomo et al., 2026a, 2026b).

### 3.1 Tick-borne encephalitis virus (TBEV)

The estimated pool prevalence of TBEV in nymphal ticks was 0.1% (range 0–0.4%), and the overall prevalence in adult ticks was 3.3% (range 0–7.1%) (Table 3). Notably, no TBEV was detected in ticks from location 1 on the southwest coast or location 4 on Hille Island in the south. In total, 7/11 RT-PCR positive samples were confirmed positive by pyrosequencing.

**Table 3:** Tick-borne encephalitis virus (TBEV) in nymphal and adult *Ixodes ricinus* ticks.

Location (year)	# of nymph pools	# of adults	Positive by RT-PCR nymphs (adults)	Positive by pyro nymphs (adults)	EPP % (CI)	MIR %	Adult prevalence % (CI)
Tungesvik 1 (2014)	31	21	0 (1)	0 (0)	0.0 (0.0-0.6)	0.0	0.0 (0.0-15.5)
Tungesvik 2 (2014)	54	29	3 (3)	2 (2)	0.4 (0.1-1.2)	0.4	6.9 (1.9-22.0)
Tungesvik 3 (2014)	78	28	2 (2)	1 (2)	0.1 (0.0-0.6)	0.1	7.1 (2.0-22.6)
Hille 4 (2014)	74	42	0 (0)	0 (0)	0.0 (0.0-0.3)	0.0	0.0 (0.0-8.4)

Overall	237	120	5 (6)	3 (4)	0.1 (0.0-0.3)	0.1	3.3 (1.3-8.3)

Pool size: 10. Pyro: pyrosequencing. EPP: Estimated Pool Prevalence. Minimum Infection Rate. CI: 95% confidence interval.

The geographical distribution of tick-borne encephalitis virus (TBEV) follows the geographical distribution of *I. ricinus* in Norway (Vikse et al., 2020). The prevalence of TBEV reported in the present study is in accordance with the findings of Vikse et al. (2020), who examined 3404 adult ticks and 44,000 nymphs of questing *I. ricinus* from 62 sites along the Norwegian coast from the Swedish border in the southeast to Brønnøy municipality in the northwest, with an overall TBEV prevalence of 0.3% in nymphs and 4.3% in adults. Similar results have been reported in several other studies from different geographical areas in Norway (Andreassen et al., 2012; Paulsen et al., 2015; Kjelland et al., 2018; Soleng et al., 2018; Lamsal et al., 2023a; Cotes-Perdomo et al., 2026a). Lamsal et al. (2023a) reported an overall estimated TBEV prevalence of 0.1% in questing *I. ricinus* nymphs in southern Scandinavia with a region-specific prevalence of 0.1% in Denmark, 0.2% in southern Sweden and 0.1% in southeastern Norway. Pettersson et al. (2014) reported an overall prevalence of 0.3% in ticks from Sweden, Finland, Denmark and Norway. In our study, the prevalence of TBEV was higher in adult ticks compared to nymphs. This is most probably due to the higher number of previous bloodmeals in adults than in nymphs. Since ticks acquire the virus primarily through a blood meal, the risk of infection increases with number of blood meals (Süss et al., 1999). In the present study, we detected TBEV in two of the three investigated locations on the west coast. These sites (site 2 and 3) are only 50 m apart. The third site, were no TBEV was detected (site 1), is located approximately 800 m from these two sites. Thus, the results might demonstrate the patchy distribution of TBEV in nature as described by Süss et al. (1999) and Chitimia-Dobler et al. (2019). The results may also be due to a low number of included ticks (310) at this site. According to the Epitool calculator for sample size with fixed pool and perfect test, it is necessary to analyze 740 nymphs (i.e. 74 pools) to expect to find one TBEV positive pool when the estimated prevalence for the region is 0.3% (Cowling et al., 1999; Ebert et al., 2010; Sergeant, 2025). However, at location 4 (the island Hille) 74 pools were examined with no findings of TBEV. Unpublished results have previously demonstrated the presence of TBEV in ticks sampled at this Island, and Paulsen et al. (2019) reported TBEV in cow milk from this island. Thus, our results indicate not only the patchy distribution of TBEV, but also variations from year to year. This notion is supported by a previous study that demonstrated variations of TBEV in questing ticks within and between sampling sites, counties and years (Vikse et al., 2020).

### 3.2 Louping-ill virus (LIV)

In the present study none of the 2490 ticks were found to be positive for LIV. There is a paucity of data on the prevalence of LIV in Scandinavia. However, LIV has been detected in ticks from the island Bornholm in Denmark (Jensen et al., 2004). In the 1980's there was an outbreak of louping-ill at a sheep farm on the southwestern coast of Norway where site 1 is located. Our finding is consistent with previous investigations that failed to detect LIV positive ticks from eastern, western, and northern Norway (cf. Paulsen et al., 2018a). This is also in accordance to clinical reports of no detection of LIV in these regions since 1991 (Gao et al., 1993; Norwegian Veterinary Institute, 2019; Ulvund et al., 1983).

The European subtype of TBEV (TBEV-Eu) and LIV are two closely related tick-borne orthoflaviviruses. However, whereas the first is the cause of one of Europe's most important zoonoses, the latter most often causes disease in sheep and red grouse (*Lagopus lagopus scotica*) (Clark et al., 2020). Clinical and histological cases of louping-ill disease were reported in sheep from the south and southwestern Norway in the 1980s (Gao et al., 1993), and the last confirmed cases was reported in 1991 (Norwegian Veterinary Institute, 2019). Louping-ill virus is believed to be maintained by ticks and mountain hare (*Lepus timidus*) (Gilbert et al., 2000; Norman et al., 2004). Ytrehus et al. (2013) reported the detection of louping-ill like flavivirus in cervids in both the southern and northwestern part of Norway. Furthermore, Ytrehus et al. (2021) found seropositive willow ptarmigan (*Lagopus lagopus lagopus*) from locations across the whole country, including areas outside the known geographical distribution of *I. ricinus*. Lamsal et al. (2023) reported ELISA seropositive TBEV but TBE neutralization negative reindeer (*Rangifer tarandus tarandus*) from three locations in Norway, also outside the geographical distribution of *I. ricinus*. However, it is not possible to distinguish between TBEV or LIV in these serological findings, and the authors concluded that the results indicate that other flavivirus, may be circulating among Norwegian semi-domesticated reindeer. Thus, either TBEV, LIV and/or another cross-reacting flavivirus infects grouse and reindeer in Norway (Ytrehus et al., 2021; Lamsal et al., 2023). This careful assessment is supported by Paulsen et al. (2020), who reported positive LIV seroreactivity in 32/38 TBE IgG positive moose and deer samples, and stated that TBEV and LIV are genetically closely related and antibodies to either virus may cross-react in the test. However, in the current study we only tested the samples molecularly by the pathogen specific molecular RT-Real time PCR methods. Of the 357 undiluted samples analysed for TBEV and LIV none of these showed any positivity PCR for LIV, only for TBEV. The positive controls, TBEV- Hochosterwitz strain and LI/Nor strain, were positive in their respective RT-Real time PCR assays. These findings are consistent with previous findings by Paulsen et al. (2018a) showing no detection of LIV in ticks at sites in eastern, western and northern Norway, and there are no clinical reports of LIV infected animals since 1991 in Norway (Gao et al., 1993; Norwegian Veterinary Institute, 2019; Ulvund et al., 1983). However, in some cases if the concentration of virus is too high this may inhibit the PCR reaction and may have an influence on the detection of the virus. Though, in this northern region of tick sampling collected from 2009 to days date the amount of virus as well as the prevalence are generally very low in nymphs (0.3%) compared to other areas in Europe (0.5–5%) (Vikse et al., 2020). Due to this low prevalence and cost efficiency we chose to analyze the samples in pools of 10, which is recommended with estimation of pooled prevalence including only one positive tick according to statistical calculations by Ebert et al. (2010) and estimated pools prevalence tool developed by AusVet (Cowling et al., 1999; Sergeant, ESG, 2018). For adult ticks the prevalence is higher than for nymphs (4.3%) but the virus concentration is similar to the nymphs. Only a handful samples in our whole history of tick analysis have high enough virus concentration (ct 25 to 27) for further analysis both in tick and human samples, usually ct value varies between 30 and 40 (Personal experience: Åshild K Andreassen).

Hence, further studies are needed to determine what viruses are circulating in this region and cross reacting in the ELISA TBE IgG analysis and affecting reindeers and willow ptarmigan.

### 3.3 *Borrelia burgdorferi* sensu lato

*Borrelia burgdorferi* s. l. was detected at all locations where adult ticks were sampled, and the overall prevalence was 5.7% (range 3.4–14.3%) (Table 4). Only 13/75 real-time positive samples were successfully sequenced. cDNA is not as suitable for these analyses as DNA (Lager et al., 2017), and likely the cause of the low success rate. To avoid problems with detection of *Borrelia* and cross reaction to tick cDNA and DNA, the cDNA was diluted prior to real-time PCR analysis. However, an underestimation of prevalence cannot be excluded.

**Table 4:** *Borrelia burgdorferi* sensu lato in adult *Ixodes ricinus* ticks.

Location (year)	# of adults	Positive by RT-PCR	Positive by sequencing	Prevalence % (CI)	# of *Borrelia afzelii*	# of *Borrelia garinii*	# of *Borrelia valaisiana*	# of *Borrelia burgdorferi* sensu stricto
Tungesvik 1 (2014)	21	7	1	4.8 (0.1-23.8)	0	1	0	0
Tungesvik 2 (2014)	29	12	1	3.4 (0.1-17.8)	0	0	0	1
Tungesvik 3 (2014)	28	3	1	3.6 (0.1-18.3)	1	0	0	0
Hille 4 (2011)	52	20	2	3.8 (0.5-13.2)	0	1	1	0
Hille 4 (2013)	0	-	-	-	-	-	-	-
Hille 4 (2014)	42	23	6	14.3 (6.8-27.2)	1	5	0	0
Hille 5 (2011)	55	10	2	3.6 (0.4-12.3)	1	1	0	0

Overall	227	75	13	5.7 (3.3-9.5)	3	8	1	1

CI: 95% confidence interval.

To be able to detect co-infections of the different bacterial pathogens in the ticks we choose to analyze adult ticks and not to analyze nymphs since they were all pools of 10 ticks. TBEV and LIV are single stranded RNA virus transcribed to cDNA and we anticipate due to the estimated low prevalence only detected one positive nymph in a pool of 10. While the bacterial pathogens like *Borrelia* have high prevalence and there may be several infected nymphs in a pool of 10. For co-infection study we therefore chose a method that could detect all bacterial pathogens within a single adult tick.

By analyzing total nucleic acid (TNA) by the MagnaPure Total Nucleic Acid High Performance Isolation kit we detected borrelia species from the mix of DNA and RNA transcribed to cDNA. With this method we hoped would improve the detection of borrelia species and other pathogens compared to other RNA extraction kits like the Qiagen RNeasy kit extraction that would extract only total RNA. Nevertheless, four genotypes of *Borrelia* were detected: *B. afzelii* (23.1%), *B. garinii* (61.5%), *B. burgdorferi* sensu stricto (7.7%) and *B. valaisiana* (7.7%).

The prevalence of *B*. *burgdorferi* s. l. is similar or lower compared to earlier studies in Norway (Mehl et al., 1987; Jenkins et al., 2001; Paulauskas et al., 2008; Kjelland et al., 2010, 2018; Soleng and Kjelland, 2013; Cotes-Perdomo et al., 2026b). In a large meta-analysis from 23 European countries the prevalence varied by region, and the average prevalence per study reached 17.8% for adult ticks (Strnad et al., 2017).

In a previous investigation conducted in 2012 where ticks were sampled at location 4, the prevalence of *B*. *burgdorferi* s. l. was reported to be 25%, compared to 14.3% in the current study (Kjelland et al., 2018). In a study conducted on the mainland, just 8.5 km east of location 4 and 5 at the island Hille, the overall prevalence of *B. burgdorferi* s. l. was 12% (Cotes-Perdomo et al., 2026b). Although the choice of nucleic acid extraction method may affect the results, it has previously been demonstrated that the prevalence varies between locations and throughout the season and between years (Jenkins et al., 2001; Kjelland et al., 2010). This shows that the current results are within the previously published range of variations. Four genotypes of *Borrelia* were detected in the present study; *B. afzelii* (n = 3), *B. garinii* (n = 8), *B. valaisiana* (n = 1) and *B. burgdorferi* sensu stricto (n = 1). However, this was based on the small subset of the samples that were successfully sequenced and may be showing a skewed variation of the genotypes. Previously, *B. afzelii* has been found to be the most frequent genospecies detected in *I. ricinus* in Norway as well as Europe (Rauter and Hartung, 2005; Kjelland et al., 2010, 2018; Soleng and Kjelland, 2013; Cotes-Perdomo et al., 2026b). Nevertheless, a higher prevalence of *B. garinii* has previously been reported at a mainland site located near locations 4 and 5 of the present study (Kjelland et al., 2010). While *B. afzelii* is typically associated with rodents, *B. garinii* and *B. valaisiana* are associated with birds (Kurtenbach et al., 2002). Further studies are needed to assess the genospecies composition on the island investigated in the current study, where it is plausible that birds serve as important tick hosts. Knowledge about the genospecies composition in a given region is essential, as the clinical manifestation of Lyme borreliosis can vary depending on the infecting *B. burgdorferi* s.l. genotype (Stanek and Strle, 2022).

### 3.4 Anaplasma phagocytophilum

The estimated pooled prevalence (EPP) in nymphs was 5.1% (range 2.1–11.1%), whereas the overall minimum infection rate (MIR) was 4.1% (range 1.9–6.9%) (Table 5). For adult ticks the overall prevalence was 19.8% (range 2.4–39.3%) (Table 5).

**Table 5:** Prevalence of *Anaplasma phagocytophilum* in *Ixodes ricinus* tick nymphs and adults.

Location (year)	Nymphs				Adults			
	# of pools	Positive by RT-PCR	EPP % (CI)	MIR %	# of adults	Positive by RT-PCR	Positive by sequencing	Prevalence % (CI)
Tungesvik 1 (2014)	31	6	2.1 (0.9-4.3)	1.9	21	6	5	23.8 (8.2-47.2)
Tungesvik 2 (2014)	54	25	6.0 (4.0-8.6)	4.6	29	4	4	13.8 (3.9-31.7)
Tungesvik 3 (2014)	78	54	11.1 (8.4-14.3)	6.9	28	11	11	39.3 (21.5-59.4)
Hille 4 (2011)	46	12	3.0 (1.6-5.0)	2.6	52	15	6	11.5 (4.3-23.4)
Hille 4 (2013)	12	3	2.8 (0.7-7.2)	2.5	0	-	-	-
Hille 4 (2014)	74	14	2.1 (1.2-3.3)	1.9	42	5	1	2.4 (0.1-12.6)
Hille 5 (2011)	26	17	10.1 (6.0-15.6)	6.5	55	19	18	32.7 (20.3-47.1)

Overall	321	131	5.1 (4.4-5.9)	4.1	227	60	45	19,8 (14.8-25.6)

Pool size: 10. EPP: Estimated Pool Prevalence. MIR: Minimum Infection Rate. CI: 95% confidence interval.

The overall prevalence of *A. phagocytophilum* appears to be relatively consistent across seven countries of the North Sea Region, although the prevalence exhibits considerable spatial variability in the range of approximately 1–5% in questing ticks (Quarsten et al., 2023). The present results confirm previously reported prevalences, ranging from 0 to 23%, from southern Norway (Radzijevskaja et al., 2008; Rosef et al., 2009). In contrast, a study in northern Norway, reported a low prevalence of 0.8% in nymphs and 4.6% in adult ticks (Soleng and Kjelland, 2013). The higher prevalence in the south-western Norway compared to in the north demonstrate the variable prevalences of *A*. *phagocytophilum* in ticks in different parts of Norway. Previously, Stuen et al. (2003) demonstrated the presence of several strains of *A. phagocytophilum* circulating simultaneously in tick-infested pastures in Norway. Even within individual sheep the various strains might have different pathogenicity (Fröhlich et al., 2023). *Anaplasma phagocytophilum* employs multiple immune evasion strategies and induces immunosuppression in the host, primarily through functional modulation of neutrophils, facilitating persistent infection (Dumler et al., 2008). Paulsen et al. (2019) indicated that co-infection with TBEV and *A. phagocytophilum* in sheep stimulates an increased TBEV antibody response, possibly also affecting the clinical outcome on infection. Anaplasmosis is not a notifiable disease in humans or animals in Norway, however, it has been estimated that 300,000 lambs are infected annually (Stuen and Bergström, 2001).

### 3.5 Wolbachia pipientis

*Wolbachia pipientis* was for detected the first time in Norway. We found this endosymbiont in all locations investigated, with an overall prevalence of 11.0% (range 5.8–25.0%) in adult *I. ricinus* ticks (Table 6).

**Table 6:** *Wolbachia pipientis* in adult *Ixodes ricinus* ticks.

Location (year)	# of adults	Positive by RT-PCR	Prevalence (CI)
Tungesvik 1 (2014)	21	4	19.0 (5.4-41.9)
Tungesvik 2 (2014)	29	2	6.9 (0.8-22.8)
Tungesvik 3 (2014)	28	7	25.0 (10.7-44.9)
Hille 4 (2011)	52	3	5.8 (1.2-15.9)
Hille 4 (2013)	0	-	-
Hille 4 (2014)	42	5	11.9 (4.0-25.6)
Hille 5 (2011)	55	4	7.3 (2.0-17.6)

Overall	227	25	11.0 (7.3-15.9)

CI: 95% confidence interval.

The origin of *Wolbachia* in ticks and its effect on tick biology is unknown, however the bacterium is known to have diverse effects on invertebrates, ranging from nutritional symbionts in nematodes to reproductive manipulators in insects (Bonnet et al., 2017). It is suggested that *Wolbachia* in *I. ricinus* ticks is transferred by the Hymenoptera endoparasitoid *Ixodiphagus hookeri* (Plantard et al., 2012). This parasitic wasp species was recorded in Norway for the first time in 2021, although it is assumed that it has been present for a longer period (Hansen and Sørlibråten, 2024). This is supported by our findings showing the presence of *Wolbachia* in ticks collected in 2011. The widespread distribution demonstrated in the present study highlights the need for further investigation to better understand the potential implications of *Wolbachia*, in ticks as well as in animals and humans.

### 3.6 Midichloria mitochondrii

*Midichloria mitochondrii* was detected at all locations, with an overall prevalence of 84.1% (range 72.4–92.9%) in adult *I. ricinus* ticks (Table 7).

**Table 7:** *Midichloria mitichondrii* in adult *Ixodes ricinus* ticks.

Location (year)	# of adults	Positive by RT-PCR	Prevalence (CI)
Tungesvik 1 (2014)	21	18	85.7 (63.7-97.0)
Tungesvik 2 (2014)	29	21	72.4 (52.8-87.3)
Tungesvik 3 (2014)	28	26	92.9 (76.5-99.1)
Hille 4 (2011)	52	39	75.0 (61.1-85.9)
Hille 4 (2013)	0	-	-
Hille 4 (2014)	42	39	92.9 (80.5-98.5)
Hille 5 (2011)	55	48	87.3 (75.5-94.7)

Overall	227	191	84.1 (78.7-88.6)

CI: 95% confidence interval.

The high prevalence of *M*. *mitichondrii* in this study is higher than previously reported by Granquist et al. (2014), who found a prevalence of 35.1% among adult ticks collected on the Norwegian west coast. Moreover, Granquist et al. (2014) found a significant difference in the prevalence of *M. mitochondrii* in nymphs between three geographical areas, ranging from 32.0% to 91.7%. *Midichloria mitochondrii* is known to be the dominant bacterium in the microbial community of *I. ricinus* (Sassera et al., 2006; Epis et al., 2013). The bacterium primarily resides in the mitochondrial intermembrane space of ovarian cells, where it replicates during the tick's blood meal and is vertically transmitted from parent to its offspring (Sassera et al., 2006, 2008; Buysse and Duron, 2018). A complete understanding of the functional basis of bacterial symbiosis and the incorporation of bacterial metabolites into tick physiology is still lacking. Studies have shown that female *I. ricinus* ticks that lack *M. mitochondrii* produce larvae that are less effective at blood-feeding compared to larvae from symbiont-infected females (Guizzo et al., 2023). This suggests that *M. mitochondrii* plays a key role in the developmental and physiological processes of *I. ricinus* ticks (Guizzo et al., 2023). Mariconti et al. (2012) found that seropositivity to *M. mitochondrii* is highly common in humans bitten by *I. ricinus* ticks (58%), in contrast to a low prevalence of 1.2% in healthy individuals not known to be bitten by ticks. These findings support the idea that *M. mitochondrii* is transmitted through tick saliva and may be infectious to vertebrates. As such, *M. mitochondrii* should be considered as a bundle of antigens introduced into the human host during a tick bite (Mariconti et al., 2012). As a result, the immune response and immune-modulation effect by *M. mitochondrii* may be important for the severeness of tick-borne infections (Bonnet and Pollet, 2021).

### 3.7 Co-occurrence of different microorganisms in adult ticks

Of the 227 adult ticks analysed, 12.3% (28) were uninfected, while 87.7% (199) harbored at least one microorganism. Among the infected ticks, 55.1% (125) carried a single pathogen, whereas 32.6% (74) exhibited co-occurrence with two or more microorganisms. Specific co-infection profiles included one tick co-infected with TBEV and *Anaplasma*, and another with the co-occurrence of *Anaplasma* and *Wolbachia*. Additionally, three ticks showed co-occurrence of TBEV and *Midichloria*, 12 with *Borrelia* and *Midichloria*, 31 with *Anaplasma* and *Midichloria*, and 16 with *Wolbachia* and *Midichloria*. Up to three microorganisms were detected in a single tick. Notably, in two ticks we found *Anaplasma*, *Borrelia*, and *Midichloria*; in one we found *Borrelia*, *Wolbachia*, and *Midichloria*; and in six we found *Anaplasma*, *Wolbachia*, and *Midichloria*. It is noteworthy that co-infection might result in increased severity of tick-borne diseases (Thomas et al., 2001; Paulsen et al., 2019; Skinner et al., 2022). Co-occurrence of endosymbionts and other pathogens may affect both the immune response, disease risk, as well as antipathogenic effects, replication effects (Skinner et al., 2022; Köhler et al., 2025). Environmental factors may play a crucial role in shaping the bacterial community in ticks which differ based on the climate, season, geographical location, available tick hosts, tick life stage and feeding status (Moreno et al., 2006; Clay et al., 2008; Steiner et al., 2008; van Overbeek et al., 2008; Carpi et al., 2011; Lalzar et al., 2012; Menchaca et al., 2013; Williams-Newkirk et al., 2014; Zhang et al., 2014; Zolnik et al., 2016).

## 4 Conclusion

Further investigations are warranted to elucidate the occurrence and epidemiological significance of co-infections and co-occurring microorganisms in ticks, and to assess the role of endosymbionts in modulating pathogens of relevance to human and animal health. Consequently, ticks represent a compelling yet challenging system for investigating the composition of their bacterial and viral communities and the associated functional and ecological implications, underscoring the need for increased research attention.

## CRediT authorship contribution statement

**Arnulf Soleng:** Conceptualization, Resources, Supervision, Visualization, Writing – original draft, Writing – review & editing. **Deepa Gurung:** Data curation, Formal analysis, Methodology, Writing – review & editing. **Vivian Kjelland:** Conceptualization, Funding acquisition, Methodology, Resources, Supervision, Writing – review & editing. **Katrine M. Paulsen:** Resources, Supervision, Writing – review & editing. **Benedikte N. Pedersen:** Resources, Writing – review & editing. **Erik G. Granquist:** Conceptualization, Methodology, Supervision, Writing – review & editing. **John H.-O. Pettersson:** Resources, Supervision, Writing – review & editing. **Rose Vikse:** Resources, Visualization, Writing – review & editing. **Snorre Stuen:** Conceptualization, Methodology, Resources, Supervision, Writing – review & editing. **Åshild K. Andreassen:** Conceptualization, Funding acquisition, Methodology, Project administration, Resources, Supervision, Visualization, Writing – review & editing.

## Conflict of interest

The authors declare that they have no known competing financial interests or personal relationships that could have appeared to influence the work reported in this paper. The study was funded by EU Interreg V-A regional program Scandtick Innovation project (grant number 20200422) and The Norwegian Ministry of Health and care Services, Barentsregion program (grant number B1412 and B1710).
